# Emotional distress in young adults during the COVID-19 pandemic: evidence of risk and resilience from a longitudinal cohort study

**DOI:** 10.1017/S003329172000241X

**Published:** 2020-06-23

**Authors:** Lilly Shanahan, Annekatrin Steinhoff, Laura Bechtiger, Aja L. Murray, Amy Nivette, Urs Hepp, Denis Ribeaud, Manuel Eisner

**Affiliations:** 1 Jacobs Center for Productive Youth Development, University of Zurich, Zurich, Switzerland; 2 Department of Psychology, University of Zurich, Zurich, Switzerland; 3 Department of Psychology, University of Edinburgh, Edinburgh, UK; 4 Department of Sociology, Utrecht University, Utrecht, The Netherlands; 5 Integrated Psychiatric Services Winterthur-Zürcher Unterland, Winterthur, Switzerland; 6 Institute of Criminology, University of Cambridge, Cambridge, UK

**Keywords:** COVID-19, distress, mental health, resilience, stress, young adults

## Abstract

**Background:**

The coronavirus disease 2019 (COVID-19) pandemic and associated lockdown could be considered a ‘perfect storm’ for increases in emotional distress. Such increases can only be identified by studies that use data collected *before* and *during* the pandemic. Longitudinal data are also needed to examine (1) the roles of previous distress and stressors in emotional distress during the pandemic and (2) how COVID-19-related stressors and coping strategies are associated with emotional distress when pre-pandemic distress is accounted for.

**Methods:**

Data came from a cohort study (*N* = 768). Emotional distress (perceived stress, internalizing symptoms, and anger), COVID-19-related stressors, and coping strategies were measured during the pandemic/lockdown when participants were aged 22. Previous distress and stressors were measured before COVID-19 (at age 20).

**Results:**

On average, participants showed increased levels of perceived stress and anger (but not internalizing symptoms) during the pandemic compared to before. Pre-COVID-19 emotional distress was the strongest predictor of during-pandemic emotional distress, followed by during-pandemic economic and psychosocial stressors (e.g. lifestyle and economic disruptions) and hopelessness, and pre-pandemic social stressors (e.g. bullying victimization and stressful life events). Most health risks to self or loved ones due to COVID-19 were not uniquely associated with emotional distress in final models. Coping strategies associated with reduced distress included keeping a daily routine, physical activity, and positive reappraisal/reframing.

**Conclusions:**

In our community sample, pre-pandemic distress, secondary consequences of the pandemic (e.g. lifestyle and economic disruptions), and pre-pandemic social stressors were more consistently associated with young adults' emotional distress than COVID-19-related health risk exposures.

From a psychological perspective, pandemics constitute life events associated with uncertainty, ambiguity, and loss of control, each of which is known to trigger stress and emotional distress, including internalizing symptoms (anxiety and depression), and anger (Ensel & Lin, 1991; Pearlin, Lieberman, Menaghan, & Mullan, 1981). The coronavirus disease 2019 (COVID-19) pandemic/lockdown are characterized by all of these features, as well as worries about one's own health and that of loved ones, economic disruption and losses (Forbes & Krueger, 2019; Frasquilho et al., 2016), lifestyle disruptions, social isolation, and loneliness (Cacioppo, Hawkley, & Thisted, 2010). Together, these conditions could create a ‘perfect storm’ for inducing emotional distress (Reger, Stanley, & Joiner, 2020).

Research on previous epidemics involving quarantines has documented declines in psychological health (for a review, see Brooks et al., 2020); studies documenting distress during the COVID-19 pandemic are rapidly emerging (e.g. de Quervain *et al*. 2020; Wang *et al*. 2020). However, these studies are typically based on cross-sectional study designs, which cannot discern whether distress has increased beyond pre-pandemic levels. Longitudinal designs with assessments *before* and *during* the pandemic are needed to examine increases in distress and the role of stressors during the pandemic when previous emotional distress is accounted for. Extant COVID-19 research has also primarily relied on convenience samples and, thus, may over-represent distressed individuals and certain demographics (e.g. females, de Quervain *et al*. 2020; Veer *et al*. 2020). Therefore, findings may not be representative of larger populations.

The current study draws on a prospective-longitudinal cohort study with data on a community-representative sample of young adults before and during the pandemic/lockdown during spring 2020. Young adults face many normative transitions (Arnett, 2000; Shanahan, 2000), which are known to be stressful (Duffy, Twenge, & Joiner, 2019), including in their educational and professional development (e.g. important exams, entry into the labor market, financial pressures, and uncertainties), social and romantic relationships, and changes in their living situation (e.g. living away from family for the first time). These normative changes and pressures could be compounded by COVID-19-related stressors and disruptions (e.g. declining labor market and inability to socialize with friends or romantic partners). Despite these potential stressors, young adults have a relatively low risk of health complications from COVID-19, are competent in using social media to connect with others, and typically do not have caregiving duties (e.g. for children or elderly parents). Thus, they also have the potential to experience resilience (i.e. adaptive or better-than-expected outcomes despite the presence of significant risk/adversity, Masten, 2001; Werner, 1993) during the COVID-19 pandemic.

During- and pre-pandemic emotional distress assessed in our study includes perceived stress, internalizing symptoms, and anger. In addition, pre-pandemic stressors typically associated with such distress, including social isolation, victimization experiences, and stressful life events, were measured. We also assessed low self-rated health to gauge participants' pre-pandemic health status. During-pandemic putative stressors assessed included one's own health risk status and that of loved ones. In addition, we assessed stressors related to secondary consequences of the pandemic (e.g. economic and lifestyle disruptions); and also hopelessness, low trust in societal responses to the pandemic, and frequent COVID-19-related news-seeking as factors that could be associated with increased emotional distress. We also assessed potentially adaptive coping strategies that could mitigate during-pandemic distress.

Data were collected in Switzerland's largest city, Zurich, which is located approximately 3 h by car/train from northern Italy, the first epicenter of the European COVID-19 outbreak. Following Italy, Switzerland was among the first European countries affected by COVID-19, ranking among the 10 most affected countries worldwide in March 2020, with one of the highest per-capita rates of COVID-19 infections (Salathe et al., 2020). The Swiss national lockdown policies were strictest from 16 March to 26 April 2020. Schools, universities, and all non-essential stores were closed, social distancing measures were enforced, social gatherings of more than five people were prohibited, working from home was implemented whenever possible, and public transport was considerably reduced (Swiss Federal Office of Public Health, 2020). Borders with neighboring countries were mostly closed. By the end of data collection (18 April 2020), Switzerland (with a population of 8.65 million) had reported 27 404 cases of COVID-19 and 1368 deaths (Worldometer, 2020). However, the case reports represent underestimates as testing was sometimes limited to at-risk individuals. Universal health care and unemployment benefits are available in Switzerland, and the government subsidized furlough schemes to prevent widespread unemployment during the pandemic.

## Methods

### Sample and procedures

Data came from the Zurich Project on the Social Development from Childhood to Adulthood (*z-proso*), a prospective-longitudinal study. The cohort comprises participants who entered first grade in one of 56 public primary schools in Zurich in 2004. The initial target sample of schools was selected using random sampling procedures (slightly oversampling disadvantaged school districts). The original study consists of eight assessment waves, at ages 7, 8, 9, 11, 13, 15, 17, and 20 (in 2018), respectively (for additional details on the sample and attrition, see, Eisner, Malti, & Ribeaud, 2011; Eisner, Murray, Eisner, & Ribeaud, 2019). In April 2020, all age 20 participants (then aged 22) were invited to participate in a COVID-19 online study. The current analysis uses stressor and emotional distress data from the age 20 and COVID-19 assessments (see online Supplementary Fig. S1).

Out of 1180 eligible participants from the age 20 assessments, 21 could not be reached due to invalid contact information/unclear status. Out of 1159 cases contacted, 786 participants responded (67.8% of age 20 sample). Due to this attrition, sampling weights were used in all analyses to allow generalizations back to the original recruitment population from 2004 (for the creation of these weights, see Nivette et al., 2020).

At age 20, participants completed surveys (lasting ~70 min) at a university research laboratory. Participants received a ~$75 cash compensation for their time. At age 22, data collection began during week 4 of the Swiss national lockdown (11 April 2020) and ended 7 days later. The online survey took ~15–20 min to complete; participants were entered into a lottery to win one of 50 prizes of ~$100. Participants provided written informed consent to participate in the study at ages 13–20 and online informed consent at age 22. Ethical approval was obtained by the Ethics Committee of the Faculty of Arts and Social Sciences of the University of Zurich. The authors assert that all procedures contributing to this work comply with the ethical standards of the relevant national and institutional committees on human experimentation and with the Helsinki Declaration of 1975, as revised in 2008.

### Measures

Below, we list the measures, and their sources and time frames. Individual items of all non-demographic measures, and their scales, scoring, and Cronbach's *α* can be found in the Online Supplement (Table S1).

#### Dependent variables (age 22)

*Perceived stress* during the past 2 weeks was assessed using four items from the Perceived Stress Scale (Cohen, Kamarck, & Mermelstein, 1983). *Internalizing symptoms* were assessed using 13 items from the Social Behavior Questionnaire (Murray, Obsuth, Eisner, & Ribeaud, 2019b) addressing depressive and anxiety symptoms in the past 2 weeks and two additional items assessing suicidal ideation and self-injury. *Anger* during the past 2 weeks was assessed using three items from the PROMIS^®^ Emotional Distress – Anger – Short Form (Pilkonis et al., 2011).

Perceived stress was correlated with internalizing symptoms and anger at *r* = 0.69 and 0.63, respectively; internalizing symptoms were correlated with anger at *r* = 0.72. Nevertheless, each construct captures somewhat different aspects and stages of emotional distress. For example, perceived stress may precede the manifestation of internalizing symptoms, whereas anger/irritability is an expression of emotional distress but is typically not well-captured in anxiety and depression scales (Vidal-Ribas, Brotman, Valdivieso, Leibenluft, & Stringaris, 2016). Therefore, we examined the correlates of each of these indicators separately.

#### Independent variables

*Family socioeconomic status* (*SES*) was assessed using the International Socioeconomic Index of occupational status (ISEI, Ganzeboom, DeGraaf, Treiman, and De Leeuw, 1992); the highest ISEI recorded for each household between child ages 7 and 15 was used. *Education/occupation* (*age 20*) was based on participants' highest educational degree and their current educational/occupational status. Categories included (1) college-track credentials or higher educational degree (*high*), (2) vocational/compulsory education, currently in education/training or employed (*medium*), (3) completion of compulsory school degree or preparatory vocational bridge year but currently not in education, employment, or training (NEET, Bynner and Parsons, 2002) (*low*). *Migration background* indicated whether both parents were born abroad (*v.* at least one parent born in Switzerland). *Living alone* was coded positive if participants did not share a household with another person at age 22.

##### Antecedent risk factors (age 20)

*Perceived stress*, *internalizing symptoms*, and *anger* at age 20 were assessed as for age 22, except with a 1-month time frame.

*Perceived social exclusion* was assessed with six items (Bude & Lantermann, 2006). *Low social support* from adults was assessed using four items created by the study team. *Bullying victimization* during the past year was assessed with four items (Murray et al., 2019a). *Low generalized trust* was measured with three items (Inglehart et al., 2014). *Low self-rated health* was measured with one item. *Stressful life events* assessed 28 potentially stressful events since the age 17 assessment. A cumulative sum score was created to capture the overall stressor load from life events.

##### Concurrent risk factors

*Health risks during COVID-19* were measured by asking respondents whether they or a loved one (e.g. family member, partner) had an occupation or a pre-existing health condition that increased their health risks during the COVID-19 pandemic. We also assessed symptoms of COVID-19, positive COVID-19 test, hospitalization because of COVID-19, and death of a loved one from COVID-19. Based on these items, six binary variables indicated the presence or absence of occupational risk, health risk, and actual illness of a loved one or oneself.

*Lifestyle disruptions* were assessed by having participants rate the degree to which COVID-19 had disrupted their lives (e.g. daily routines, work, education, and family). *Economic disruption* was assessed by asking participants whether they had financial problems due to the current situation. *Loss of occupation*/*education* assessed job loss, suspension of educational program, or problems with one's business during the COVID-19 outbreak. *Hopelessness* was assessed with one item. *Low trust in society's responses to COVID-19* was measured using six items assessing the degree to which respondents distrusted the government's responses to, other people's responses to, and media coverage about the COVID-19 crisis.

*Frequent COVID-19 news-seeking* was assessed by asking respondents how often during the day they sought news or information about COVID-19.

#### Variables used in follow-up analyses

*Coping*. We assessed several coping strategies, including emotional support-seeking, self-distraction, acceptance, and positive reappraisal/reframing, with one item each, adapted from Carver (1997). In addition, several coping strategies that may have been particularly important during the COVID-19 lockdown (keeping a daily routine, physical activity/exercise, helping others, and seeking professional mental health support) were assessed.

*Relative change in well-being*. Respondents rated the extent to which they currently felt worse or better compared to before the COVID-19 pandemic using a 10-point scale. Based on this scale, we coded a categorical variable: *feeling worse* (1–4), *feeling approximately the same* (5–6), and *feeling better* (7–10).

*Open-ended comments*. In a final open-ended comments section, participants were invited to share any additional thoughts about the COVID-19 crisis and their current well-being.

### Analytic strategy

Paired sample *t* tests were used to compare absolute levels of pre- and during-pandemic emotional distress. Regression analyses were performed in separate steps to examine the antecedents and concurrent correlates of during-pandemic emotional distress. First, we analyzed associations of pre- and during-pandemic stressors/risks with during-pandemic levels of emotional distress. For this purpose, each pre- and during-pandemic correlate was entered separately while adjusting for sociodemographic characteristics only. Second, we analyzed whether pre- and during-pandemic stressors and risk factors were associated with change in individual differences in distress. For this purpose, the lag of the outcome at the previous time point was added to examine predictors of COVID-19 distress ‘net’ of pre-pandemic distress. Third, all demographic variables and all significant concurrent correlates from the previous step were entered into one model, keeping only significant predictors. Fourth, in a separate model, the same step was repeated for all antecedent predictors. Thus, the third and fourth steps resulted in trimmed models of final concurrent and antecedent correlates.

Attrition analyses showed that, compared to the first assessment at age 7, respondents in the age 22 COVID-19 survey were more likely to be female and from a non-migrant background (*p* < 0.001 and *p* = 0.006, respectively). The percentage of missing data in each assessment was low. Nevertheless, we used multiple imputation to address any potential bias (Enders, 2017; Schafer & Graham, 2002). We specified an imputation model with all variables used in our study; 20 imputed data sets were generated. Multiple regression analyses were then performed in *MPlus* (Muthén & Muthén, 2012) to examine the antecedents and correlates of distress during COVID-19. We estimated linear models (in which all outcome variables were continuous) using the maximum likelihood robust estimator. Parameter estimates were averaged across the imputed data sets and standard errors were pooled following Rubin's rules (Rubin, 1987).

## Results

Table 1 shows the descriptive statistics for all study variables. Paired sample *t* tests revealed that young adults' mean perceived stress levels and anger were higher during the pandemic compared to the pre-pandemic assessment (*p* < 0.001). The mean of internalizing symptoms decreased (*p* < 0.001). Only a minority of participants worked in an occupation that increased their risk of contracting COVID-19, had a health condition that increased their risk of COVID-19 complications, or had experienced symptoms of or were diagnosed with or hospitalized for COVID-19. Most participants had a loved one working in an at-risk occupation or with a health condition that increases their risk of complications, but only a minority of participants had a loved one who had either been diagnosed or hospitalized with COVID-19 or had died from it. On average, participants rated the COVID-19 crisis as somewhat disruptive to their lifestyle (i.e. daily routine, work, education, and family). Approximately one in seven participants reported economic disruption. More than one in five reported frequent news-seeking in relation to COVID-19. Online Supplementary Table S2 shows descriptive variables by sex, revealing, for example, that females reported higher levels of pre- and also during-pandemic emotional distress compared to males on all indicators. Females also reported higher levels of during-pandemic lifestyle disruptions and hopelessness than males.

**Table 1.** Descriptive statistics for all study variables (based on weighted sample)

	%	*N*	*M*	s.d.
Outcomes at age 22, COVID-19				
Perceived stress (range: 1–5)			2.91	0.92
Internalizing symptoms (range: 1–5)			2.00	0.69
Anger (range: 1–5)			2.59	0.94
Outcomes at age 20, pre-COVID-19				
Perceived stress (range: 1–5)			2.79	0.95
Internalizing symptoms (range: 1–5)			2.12	0.74
Anger (range: 1–5)			2.37	0.75
Sociodemographics and living situation				
Female	48.1	378		
Family ISEI (range: 10–90)			50.55	19.74
Migration background (1 = both parents born abroad)	50.9	394		
Education (age 20)				
Low (NEET)	2.2	17		
Medium	69.6	546		
High	28.2	221		
Living alone (age 22)	5.4	42		
Stress and health before COVID-19 (age 20)				
Perceived social exclusion (range: 1–4)			1.49	0.58
Low social support (range: 1–4)			1.76	0.68
Bullying victimization (range: 1–6)			1.36	0.46
Low trust (range: 1–4)			2.65	0.67
Sum of stressful life events in previous 3 years (range: 1–28)			6.64	3.15
Low self-rated health (range: 0–100)			43.76	22.88
Health risks during COVID-19 (age 22)				
Occupational risks – loved one	54.9	429		
Health risks – loved one	57.3	448		
Actual illness – loved one	14.1	110		
Occupational risks – self	26.5	207		
Health risks – self	11.5	90		
Actual illness – self	24.4	191		
Stressors during COVID-19 (age 22)				
Lifestyle disruptions (range: 1–10)			6.28	2.44
Economic disruption	14.4	112		
Loss of occupation/education	6.6	52		
Hopelessness (range: 1–10)			4.12	2.03
Low trust in society's response (range: 1–4)			2.05	0.47
Frequent COVID-19 news-seeking	28.6	225		

ISEI, International Socioeconomic Index of occupational status; NEET, not in education, employment, or training.

**Fig. 1.**
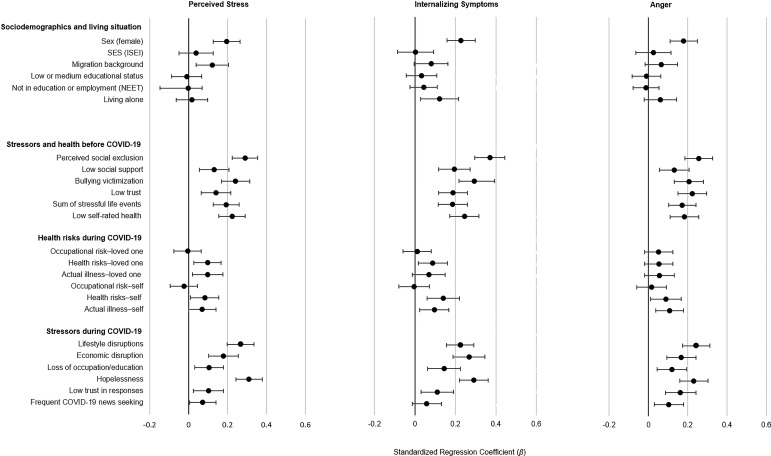
Associations of sociodemographic and risk variables with *levels of* emotional distress during the COVID-19 pandemic/lockdown. Models that used stressors and health risks as predictors were adjusted for all sociodemographic variables. Risk factors were entered one at a time (i.e. a separate model for each risk factor). Standardized regression coefficients (*β*) and 95% confidence intervals (CIs) were applied. For exact coefficients, CIs, and *p* values, see online Supplementary Table S3.

Figure 1 shows associations between each correlate and each outcome, adjusting for sociodemographic variables; these coefficients of concurrent correlates of during-pandemic emotional distress could be compared to those from other cross-sectional work (for exact coefficients and *p* values, see online Supplementary Table S3). Females were at higher risk of each of the three emotional distress indicators. Having a migrant background was associated with more perceived stress. In addition, pre-pandemic social stressors, stressful life events, low generalized trust, poor self-rated health, and concurrent pandemic-related stressors (i.e. during-pandemic lifestyle and economic disruptions, loss of occupation/education) and other risks (e.g. hopelessness and low trust in responses) were associated with during-pandemic distress. Frequent news-seeking was associated with perceived stress and anger. Health risks to self and loved ones during the pandemic generally had small or no associations with distress.

**Fig. 2.**
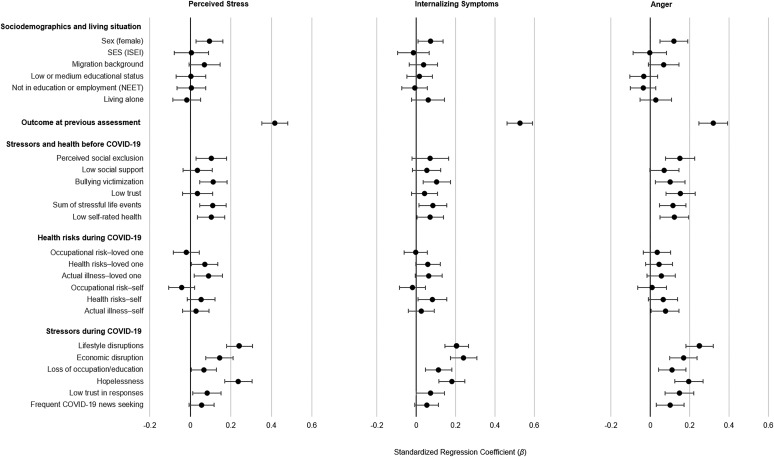
Associations of sociodemographic and risk variables with *changes in* emotional distress from the pre-pandemic to the during-pandemic/lockdown assessment (i.e. adjusted for pre-pandemic distress). Models that used stressors and health risks as predictors were adjusted for all sociodemographic variables. Risk factors were each entered one at a time (i.e. a separate model for each risk factor). Standardized regression coefficients (*β*) and 95% CIs were applied. For exact coefficients, CIs, and *p* values, see online Supplementary Table S4.

Figure 2 shows results of the analyses in which all models depicted in Fig. 1 were adjusted for previous distress (i.e. adjusted for the outcome variable at age 20; for exact coefficients and *p* values, see online Supplementary Table S4). Thus, the coefficients for risk factors depicted indicate risk of greater increases in perceived stress and anger during the pandemic assessment compared to before relative to others in the sample (or fewer decreases in internalizing symptoms relative to others as internalizing symptoms decreased on average).

Those with previous emotional distress were at considerably increased risk of during-pandemic emotional distress; internalizing symptoms had the highest stability among the distress indicators. With the inclusion of previous emotional distress the size of the coefficient for female sex was reduced by about half. The inclusion of previous distress reduced the size of some associations between pre-pandemic stressors (e.g. low social support) and during-pandemic distress, but pre-pandemic bullying victimization, stressful life events, perceived social exclusion, and low self-rated health still predicted pre- to during-pandemic increases in emotional distress. Many during-pandemic/lockdown stressors, including lifestyle and economic disruptions and loss of education or employment, were associated with greater increases in emotional distress. In addition, hopelessness was associated with during-pandemic distress. Indeed, after pre-pandemic distress, during-pandemic stressors and hopelessness were the strongest correlates of during-pandemic distress. Health risks to or actual COVID-19 illness of loved ones were associated with increases in perceived stress; being in the health risk group was associated with internalizing symptoms, and having had symptoms or a diagnosis of or having been hospitalized for COVID-19 was associated with anger. All other associations between the health risk variables and emotional distress were not significant.

Table 2 shows associations from the final multivariate models which aimed to understand which correlates explained unique variance in during-pandemic emotional distress when taking into account pre-pandemic emotional distress and other significant correlates at the same time point. Pre-pandemic distress and lifestyle and economic disruptions and hopelessness during the pandemic were most strongly associated with during-pandemic perceived stress, internalizing symptoms, and anger. Pre-pandemic bullying victimization and cumulative stressful life events were also uniquely associated with during-pandemic emotional distress. Some correlates were associated with a single emotional distress outcome only. For example, migration background and a loved one's COVID-19 health risks or actual illness were (weakly) associated with increases in perceived stress only. Furthermore, low pre-pandemic generalized trust, low concurrent trust in society's responses, and frequent COVID-19-related news-seeking were associated with increases in anger only. With the inclusion of during-pandemic lifestyle disruptions and hopelessness, the sex coefficient was reduced considerably (to non-significance for perceived stress and internalizing symptoms). Table 2 shows that there were no correlates associated with internalizing symptoms only.

**Table 2.** Results from final trimmed models (estimated separately for concurrent and antecedent predictors)

Perceived stress	Internalizing symptoms	Anger
Demographics and concurrent correlates • Hopelessness (*β* = 0.21, *p* < 0.001) • Lifestyle disruptions (*β* = 0.20, *p* < 0.001) • Economic disruption (*β* = 0.09, *p* = 0.009) • Actual illness – loved ones (*β* = 0.08, *p* = 0.014) • Migration background (*β* = 0.08, *p* = 0.035) • Health risks – loved ones (*β* = 0.06, *p* = 0.068)	Demographics and concurrent correlates • Economic disruption (*β* = 0.20, *p* < 0.001) • Lifestyle disruptions (*β* = 0.16, *p* < 0.001) • Hopelessness (*β* = 0.14, *p* < 0.001)	Demographics and concurrent correlates • Lifestyle disruptions (*β* = 0.21, *p* < 0.001) • Hopelessness (*β* = 0.14, *p* < 0.001) • Low trust in society's response (*β* = 0.12, *p* < 0.001) • Economic disruption (*β* = 0.11, *p* = 0.003) • Frequent COVID-19 news-seeking (*β* = 0.09, *p* = 0.014) • Female (*β* = 0.09, *p* = 0.011) • Health risks – self (*β* = 0.06, *p* = 0.082) • Medium education level (*β* = −0.06, *p* = 0.084, ref = high education)
Antecedent predictors • Previous perceived stress (*β* = 0.34, *p* < 0.001) • Stressful life events (*β* = 0.10, *p* = 0.003) • Bullying victimization (*β* = 0.09, *p* = 0.009) • Low self-rated health (*β* = 0.08, *p* = 0.018)	Antecedent predictors • Previous internalizing (*β* = 0.47, *p* < 0.001) • Bullying victimization (*β* = 0.10, *p* = 0.007) • Stressful life events (*β* = 0.08, *p* = 0.035)	Antecedent predictors • Previous anger (*β* = 0.22, *p* < 0.001) • Low generalized trust (*β* = 0.11, *p* = 0.004) • Perceived social exclusion (*β* = 0.10, *p* = 0.012) • Stressful life events (*β* = 0.09, *p* = 0.011) • Low self-rated health (*β* = 0.08, *p* = 0.036)

*Note*: Each final model included all demographic variables and the respective outcome at the previous assessment. For the upper row of results, all significant concurrent correlates from Fig. 2 were entered simultaneously; those with *p* ⩾ 0.10 were trimmed. For the lower row of results, all significant antecedent correlates from Fig. 2 were entered simultaneously; those with *p* ⩾ 0.10 were trimmed. Results for demographic factors and previous emotional distress are shown only once to avoid redundancy. Concurrent and antecedent predictors are ordered by size of the standardized regression coefficient.

### Follow-up analyses

#### Coping strategies

Online Supplementary Table S5 shows descriptive statistics for all coping variables. There are several possible processes that may underlie associations between young adults' use of coping strategies and emotional distress: First, individuals who are distressed by the pandemic/lockdown may more frequently use certain coping strategies (resulting in positive coping–distress associations). Second, frequent use of other coping strategies may work more preventatively or instantaneously (resulting in negative coping–distress associations). Consistent with the first process, Table 3 shows that several coping strategies, specifically seeking social support, engaging in distractions, and seeking professional help, were used more frequently by those with more pandemic/lockdown distress. Consistent with the second process, frequent use of several other coping strategies, specifically keeping a daily routine, positive reappraisal/reframing, engaging in physical activity, acceptance, and keeping in contact with family and friends, was associated with reduced distress (the latter two were associated with reduced internalizing problems only). Given that coping strategies were assessed at the same time as the emotional distress measures, additional processes, including bidirectional processes, may also be consistent with these findings.

**Table 3.** Associations between coping strategies and emotional distress during COVID-19

		Perceived stress			Internalizing symptoms			Anger	
	*β*	95% CI	*p*	*β*	95% CI	*p*	*β*	95% CI	*p*
Emotional support-seeking	**0.16**	0.09–0.23	<0.001	**0.14**	0.07–0.21	<0.001	**0.17**	0.10–0.24	<0.001
Self-distraction	**0.17**	0.10–0.24	<0.001	**0.14**	0.08–0.20	<0.001	**0.16**	0.09–0.23	<0.001
Acceptance (of COVID-19 crisis)	−0.04	−0.11 to 0.03	0.277	**−0.08**	−0.15 to −0.01	0.021	−0.07	−0.16 to 0.01	0.076
Positive reappraisal/reframing	−0.06	−0.13 to 0.01	0.071	**−0.15**	−0.22 to −0.08	<0.001	**−0.15**	−0.22 to −0.08	<0.001
Physical activity/exercise	**−0.12**	−0.19 to −0.05	0.001	**−0.10**	−0.17 to −0.04	0.002	**−0.09**	−0.16 to −0.01	0.025
Helping others	0.01	−0.05 to 0.08	0.703	−0.01	−0.07 to 0.05	0.804	0.02	−0.04 to 0.09	0.485
Keeping in contact with family/friends	−0.04	−0.11 to 0.03	0.252	**−0.07**	−0.14 to −0.01	0.029	−0.07	−0.14 to 0.00	0.066
Keeping daily routine	**−0.17**	−0.24 to −0.10	<0.001	**−0.17**	−0.23 to −0.11	<0.001	**−0.12**	−0.20 to −0.05	0.001
Seeking professional help	**0.14**	0.07–0.21	<0.001	**0.19**	0.11–0.28	<0.001	**0.16**	0.08–0.23	<0.001

Adjusted for sociodemographic variables and emotional distress prior to the pandemic. Coping strategies were each entered one at a time (each coping strategy = a separate model). Standardized regression coefficients (*β*).

Bolded coefficients significant at *p < 0.05.

#### Feeling better *v*. worse

We directly asked participants whether they were doing better, approximately the same, or worse during the pandemic compared to before; 50.8% reported feeling approximately the same (or just slightly worse or better), 18.7% reported feeling notably better, and 30.5% reported feeling notably worse. The continuous feeling worse item was correlated with increased emotional distress during COVID-19, as measured by the differences (COVID-19 score minus the age 20 score) for the respective outcomes (*r* = 0.156, *p* < 0.001; *r* = 0.218, *p* < 0.001; and *r* = 0.231, *p* < 0.001 for correlations with increased perceived stress, internalizing symptoms, and anger, respectively). In the open-ended comments, young adults who reported feeling better most frequently cited a positive deceleration of life as a reason for feeling better. Those feeling worse most frequently reported being frustrated with society's response to the pandemic and uncertainty about the future (of the pandemic, society, and their personal educational or professional future).

## Discussion

The stress-inducing characteristics of the COVID-19 pandemic and associated lockdown – which include uncertainty, ambiguity, loss of control, social isolation, and worries about one's own health and that of loved ones – could induce or increase stress and stress-related mental health problems, including internalizing symptoms and anger (Reger et al., 2020). Although most young adults are at low risk of physical health complications from COVID-19, they may be distressed by the pandemic's secondary consequences, including the lockdown and associated social standstill and economic decline. Indeed, these secondary consequences of the pandemic could be especially troubling for young adults as they attempt to tackle many of life's key transitions (e.g. educational, professional, social, and romantic relationships, Arnett, 2000; Shanahan, 2000), but are now frustrated in these efforts.

This study leveraged a prospective-longitudinal cohort study to examine several important issues relating to the pandemic/lockdown and young people's mental health, including the roles of previous distress and stressors in during-pandemic emotional distress, which can only be examined with a combination of pre- and during-COVID-19 assessments. The largest risk factor for emotional distress during COVID-19 was previous emotional distress. Stability of stress and psychopathology is a well-known phenomenon (Copeland, Shanahan, Costello, & Angold, 2009) and should be considered for the identification of those in need of during-pandemic mental health services. In addition, pre-pandemic social stressors (e.g. bullying victimization, stressful life events, and feelings of social exclusion) predicted during-pandemic emotional distress. It is possible that the effects of pre-COVID-19 social stressors may be exacerbated during the pandemic/lockdown (e.g. by limited opportunities for social contact).

Among during-pandemic stressors, the secondary consequences of the pandemic/lockdown, including lifestyle and economic disruption, and feeling hopeless, were most strongly associated with emotional distress. This is consistent with previous work reporting that economic disruptions are accompanied by declines in mental health (Forbes & Krueger, 2019). Economic downturn changes young adults' future outlook, including their visions and hopes for their professional and economic future (Gassman-Pines, Gibson-Davis, & Ananat, 2015). Despite the availability of certain safety nets in Switzerland (e.g. unemployment benefits and furlough schemes), young adults, who are relatively new to or just transitioning into the job market, may be more likely to fall through the gaps in these safety nets. Economic disruptions also tend to be associated with tensions in interpersonal relationships, which can further impact mental well-being (Conger, Ge, Elder, Lorenz, & Simons, 1994).

Surprisingly, health risks to self or others during the COVID-19 pandemic were only weakly associated with emotional distress. This could be due to the fact that only a small percentage of participants was exposed to the most traumatic aspects of the pandemic (e.g. death of a loved one or own hospitalization due to COVID-19). Furthermore, young adults with work-related potential exposure to the virus may not perceive themselves as being at risk of serious COVID-19-related complications, and/or could have found a sense of meaning or purpose in contributing to society during the pandemic (which could increase resilience). In addition, the Swiss lockdown was effective at ‘flattening the curve’, and at no point during March or April 2020 were hospitals or intensive care units in Zurich overwhelmed. Flattening the curve may not only reduce risks to physical health, but could also have positive downstream effects on mental health.

Female young adults had a higher risk than males of pre- and also during-pandemic distress, which is consistent with previous work reporting that females are generally more prone to internalizing-spectrum symptoms (Duffy et al., 2019). Indeed, with the inclusion of previous distress, the size of the female sex coefficient in the prediction of during-pandemic distress was halved. It was further reduced considerably (to non-significance for perceived stress and internalizing symptoms) with the inclusion of during-pandemic lifestyle disruptions and hopelessness (which had higher levels for females than males). Young adults with a migration background were also at increased risk of during-pandemic perceived stress, perhaps because of separation or isolation from loved ones due to closed borders, greater likelihood to work in jobs affected by the pandemic, or worries about loved ones in heavily affected countries.

Together, our findings suggest several targets for prevention/intervention. First, females, migrants, and young adults with higher pre-pandemic emotional distress, social exclusion, and an accumulation of stressful life events may need additional mental health supports and services during pandemics and lockdowns. Second, during times of unexpected disruption, educational and professional development institutions and responsible government agencies should aim to establish clear communication with young adults and make supportive measures available – perhaps especially for young adults in the final stages of their educational and professional development. Third, supplemental income measures could alleviate distress among economically vulnerable young adults who are not covered by unemployment or furlough payments. Finally, educating young adults about select coping strategies could counteract emotional distress during a pandemic.

Indeed, our findings show that keeping a daily routine, engaging in physical activity/exercise, positive reappraisal/reframing, and additional coping strategies were associated with lower distress. The association of positive reappraisal with less emotional distress is consistent with another recent study (Veer et al., 2020). Importantly, positive reappraisal (i.e. changing thought patterns about events that cannot themselves be changed) is a skill that can be practiced and improved (Beck, 2016) through avenues such as internet-based applications (Donker et al., 2013) and online cognitive behavior therapy (Axelsson et al., 2020). Regular physical activity is known to be an effective antidepressant (Harvey et al., 2018). Switzerland did not institute home confinement, allowing individuals to exercise outside, which may have alleviated distress. Although we cannot infer causality from our cross-sectional analyses of coping and emotional distress, our results suggest actionable targets for prevention/intervention, even within the restraints of lockdowns, although these will need to be evaluated in future research. Future longitudinal during-pandemic study designs will also need to further illuminate whether increased use of certain coping strategies (e.g. emotional support-seeking) will result in decreased emotional distress over time.

Almost one in five young adults reported feeling better during than before the pandemic, a finding consistent with another recent study (de Quervain et al., 2020). This phenomenon is worth exploring considering the pre-pandemic trends of increasing stress and internalizing symptoms among contemporary Western youth in recent decades (Keyes, Gary, O'Malley, Hamilton, & Schulenberg, 2019; Twenge, Cooper, Joiner, Duffy, & Binau, 2019), and the need for measures to reverse these trends. Participants whose well-being improved during the pandemic tended to appreciate the opportunity to decelerate their life. Additional work is needed to pinpoint the specific reasons for improved well-being during pandemic/lockdown conditions with the goal of applying these to post-pandemic life. In the open-ended comments, several participants suggested that being removed from workplace or educational pressures, more time with family, partners and close friends, spending time on hobbies, and the opportunity to sleep more contributed to better well-being during the pandemic; these and additional potential causes of better during-pandemic well-being warrant future systematic investigation.

### 

#### Limitations

Our study has the important strength of including both pre- and during-COVID-19 assessments, but it also has limitations. First, symptoms of post-traumatic stress disorder were not assessed but may have increased in individuals directly or indirectly exposed to COVID-19 at-risk occupations, health risks, hospitalizations, or death of loved ones. Second, the pre-pandemic assessment occurred approximately 2 years before the COVID-19 crisis, and some of the changes observed here could have been due to typical age-related development or other stressors preceding the pandemic. Third, most stressors and life events assessed during the pandemic were COVID-19-specific. Other ongoing stressors in participants' lives could have also increased their distress during COVID-19. Fourth, coping and emotional distress were measured at the same assessment, during the pandemic, meaning that the directions of effects underlying their association are uncertain. For example, it is not clear whether distressed individuals had recruited additional emotional support or whether co-ruminating with others about COVID-19-related stressors increased emotional distress. Fifth, those acutely affected by COVID-19 (or whose loved ones were acutely affected) may not have participated in the survey. Sixth, our assessments took place in weeks 4 and 5 of the lockdown in Switzerland; findings could change with prolonged social distancing and lockdown measures, and in places where the lockdown is less successful in flattening the curve. Finally, while our sample was generally representative of young adults in the Zurich area, findings may not generalize to regions with different lockdown strategies; different rates of COVID-19 cases, hospitalizations, and deaths; or different social systems and safety nets.

## Conclusion

In our sample of young adults, economic and social factors were more strongly and consistently associated with distress during the COVID-19 crisis than exposure to virus-related health risks. Indeed, previous distress and COVID-19-related economic and lifestyle disruptions and hopelessness were among the strongest correlates of young adults' distress during the lockdown, followed by pre-pandemic victimization experiences and accumulation of stressful life events. Keeping a daily routine, physical activity and exercise, and positive reappraisal/reframing were associated with less distress, and young adults whose well-being improved during the pandemic/lockdown tended to comment on a positive deceleration of their lives. Despite its many downsides, the pandemic/lockdown may have given some young people the opportunity to take stock of their lives and to improve their long-term well-being.

## Supplementary material

For supplementary material accompanying this paper visit http://dx.doi.org/10.1017/S003329172000241X. click here to view supplementary material
