# Supplementary material for: Emotional distress in young adults during the COVID-19 pandemic: evidence of risk and resilience from a longitudinal cohort study
Source: Psychol Med. 2020 Jun 23:1–10. doi: 10.1017/S003329172000241X (PMC7338432; doi:10.1017/S003329172000241X)
Supplement: Supplementary file 1 [file S003329172000241Xsup001.docx]

**Online Supplement:**

**Emotional Distress in Young Adults During the COVID-19 Pandemic:**

**Evidence of Risk and Resilience From a Longitudinal Cohort Study**

*Lilly Shanahan, Annekatrin Steinhoff, Laura Bechtiger, Aja L. Murray, Amy Nivette, Urs Hepp, Denis Ribeaud, & Manuel Eisner*

Supplementary Figure S1. Depiction of longitudinal design of current study.


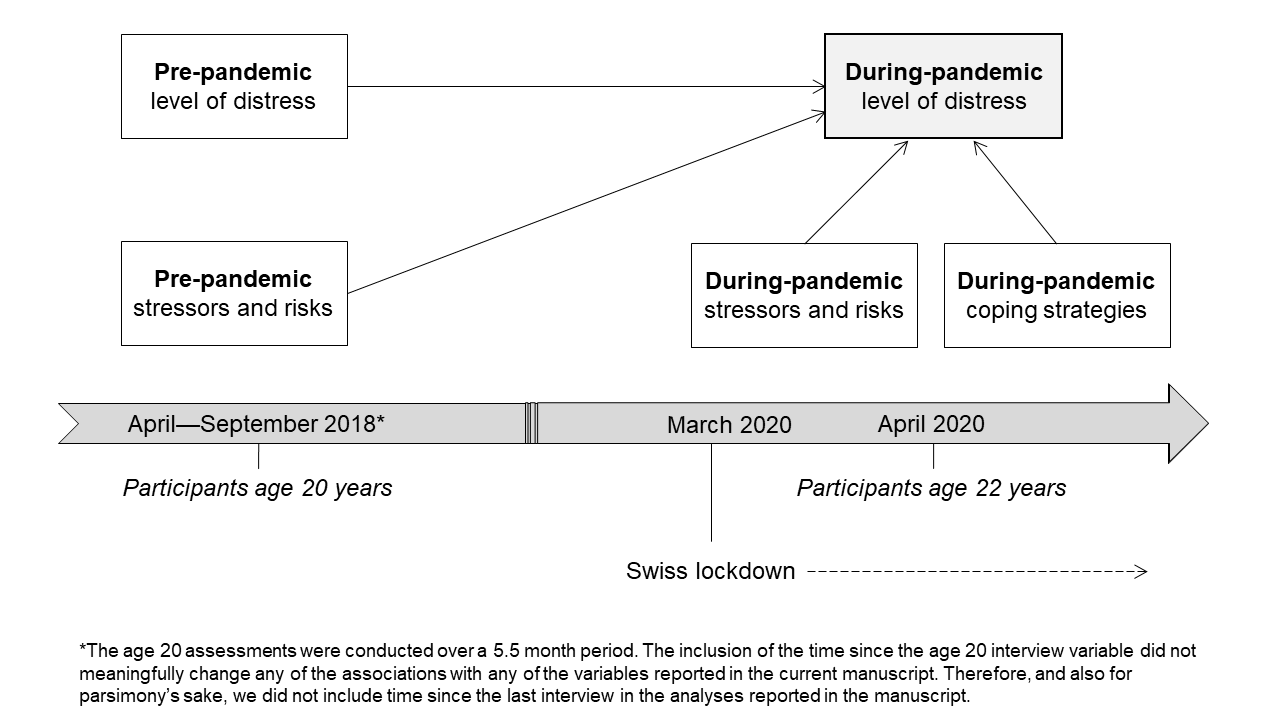


Supplementary Table S1. Detailed overview of study measures. Relevant citations can be found in the measures section of the article.

| Measure | Item(s) | Scale | Cronbach’s α | Scoring |
| --- | --- | --- | --- | --- |
| **Emotional Distress (age 20 & 22)** | | | | |
| Perceived Stress (4 items) -- from Perceived Stress Scale (Cohen, Kamarck, & Mermelstein, 1983) | During the past two weeks, how often… (age 22)  During the past month, how often… (age 20)   - … have you felt that you were unable to control the important things in your life? - … have you felt nervous and “stressed”? - … have you felt that you could not cope with all the things that you had to do? - … have you felt difficulties were piling up so high that you could not overcome them? | 1 = never to 5 = very often | 0.86 (age 22)  0.86 (age 20) | Mean score  (possible range: 1-5) |
| Internalizing Symptoms (15 items)  -- from Social Behavior Questionnaire (Murray, Obsuth, Eisner, & Ribeaud, 2019) | During the past two weeks, how often have you felt the following? (age 22)  During the past month, how often have you felt the following?  (age 20)   - I was bored. - I didn’t feel joy about anything. - I cried. - I hated myself. - I felt so tired that all I could do was sit around and do nothing. - I was scared for no particular reason. - I felt unhappy. - I felt alone. - I injured myself on purpose (e.g. cut myself, tore open wounds, hit my head, pulled out my hair). - I couldn’t fall asleep at night. - I felt like I was no good for anything. - I thought about killing myself. - I felt like I did everything wrong. - I was sad without knowing why. - I was worried. | 1 = never to 5 = very often | 0.90 (age 22)  0.92 (age 20) | Mean score  (possible range: 1-5) |
| Anger (3 items) -- from PROMIS® Emotional Distress—Anger—Short Form (Pilkonis et al., 2011) | During the past two weeks, how often have you felt the following?  (age 22)  During the past month, how often have you felt the following?  (age 20)   - I felt angry. - I felt annoyed. - I was grouchy. | 1 = never to 5 = very often | 0.78 (age 22)  0.71 (age 20) | Mean score  (possible range: 1-5) |
| Relative change in well-being from before to during COVID-19 pandemic (1 item) | How much better or worse do you feel since the beginning of the corona crisis? | 1 = extremely worse to 10= extremely better | --- | Categorical variable *feeling worse* (1-4), *feeling approximately the same* (5-6), and *feeling better* (7-10) |
| **Stressors and Health Before COVID-19 (age 20)** | | | | |
| Perceived social exclusion (6 items)  -- from Bude & Lantermann (2006) | This is about how you see yourself opposite other people and society. What do you think about the following statements?   - I feel like I’m not really part of society. - I get excluded. - I don’t have a chance in this society anyway. - I feel written off by other people. - I feel like a stranger here. - I feel surplus to society. | 1 = fully untrue to 4 = fully true | 0.88 | Mean score  (possible range: 1-4) |
| Low social support  (4 items) | How true are these statements for you?   - There are adults I can talk to about my problems. - Out of the adults that I know, there are some I admire. - I discuss my problems with adults. - There are adults I can rely on. | 1 = fully untrue to 4 = fully true (*reverse coded)* | 0.81 | Mean score  (possible range: 1-4) |
| Bullying victimization  (4 items)  -- from Murray, Eisner et al. (2019) | In the past 12 months, how often have other people purposely…   - … ignored or excluded you? - … laughed at you, mocked or insulted you? - … hit, bit, kicked you or pulled your hair? - … took away your possessions, destroyed them or hid them? | 1 = never to 6 = (almost) every day | 0.62 | Mean score  (possible range: 1-6) |
| Low (generalized) trust  (3 items)  -- from Inglehart, Haerpfer et al. (2014) | This is about how you see people in everyday life. How true are these statements for you?   - Most people can be trusted. - People usually try to help one another. - Most people try to be fair. | 1 = fully untrue to 4 = fully true (*reverse coded)* | 0.85 | Mean score  (possible range: 1-4) |
| Stressful life events in past 3 years  (28 items) | Have you experienced one of the following events in the past three years?   - You moved in with a foster family or moved to a home. - You moved away from your parents and now live independently (alone, with a partner, or in a house-share). - You were hospitalized for several days because you were ill or because you had an accident. - You spent several days in a psychiatric clinic because of a psychological illness (e.g. depression). - Your father or your mother were hospitalized for several days because they were ill or because they had an accident. - Your sister or your brother was hospitalized for several days because s/he was ill or because s/he had an accident. - A good friend of yours was hospitalized for several days because s/he was ill or because s/he had an accident. - Your grandfather or grandmother died. - Your sister or brother died. - Your mother or father or a different adult that cares for you at home died (e.g. your stepmother or your mother’s partner). - Another person you were close to died (e.g. a good friend, aunt, cousin, classmate). - Your parents divorced or separated and one of them moved away from home. - The new partner of your mother or of your father moved in with you (please also mark if you moved in with her/him). - A new brother or sister was born and now lives with you. - Your brother or sister moved away from home. - Your mother or father lost their job and became unemployed. - You had to repeat a grade. - You failed an important exam or probation period at school. - You couldn’t find an apprenticeship, even though you looked for one. - You quit your education or training or got fired from your apprenticeship. - You got a reprimand from school or you had to go to the principal because of your behavior. - You got reported to the police and were questioned by them. - You got sentenced to prison. - You broke up with/were broken up with by a romantic partner (male/female). - You broke up with your best friend or he/she did not want to be friends with you anymore. - There was a burglary at your house. - You were forced to perform or endure sexual acts under serious threat or use of force. - You were victim of a violent crime and had to be medically treated as a consequence. | 1= Yes and 0 = No | --- | Sum score  (possible range: 0-28) |
| Low self-rated health  (1 item) | In terms of my health, I feel healthier than XX of 100 people my age. | 0-100 (*reverse coded)* | --- | (possible range: 1-100) |
| **Health Risks During COVID-19 (age 22)** | | | | |
| Occupational risks–loved one  (1 item) | A family member, partner or other close person has an occupation, which increases their risk of infection. | 1= Yes and 0 = No | --- |  |
| Health risks–loved one  (1 item) | A family member, partner or other close person has a pre-existing health condition, which increases their risk during the corona virus pandemic. | 1= Yes and 0 = No | --- |  |
| Actual illness–loved one  (1 item) | A family member, partner or other close person was…   - … tested positive for COVID-19 but was not hospitalized. - … tested positive for COVID-19 and was hospitalized because of the infection. - …died due to an infection with corona virus disease. | 1= Yes and 0 = No | --- | Aggregated  (0= no item is endorsed, 1 = at least 1 item is endorsed) |
| Occupational risks–self  (1 item) | I have an occupation, which increases my risk of infection with corona virus. | 1= Yes and 0 = No | --- |  |
| Health risks–self  (1 item) | I have a pre-existing health condition which puts me especially at risk during the corona pandemic. | 1= Yes and 0 = No | --- |  |
| Actual illness–self  (1 item) | I…   - …had symptoms of the corona virus. - … tested positive for COVID-19 but was not hospitalized. - …was hospitalized because I got ill with COVID-19. | 1= Yes and 0 = No | --- | Aggregated  (0= no item is endorsed, 1 = at least 1 item is endorsed) |

| **Stressors During COVID-19 (age 22)** | | | | |
| --- | --- | --- | --- | --- |
| Lifestyle disruptions  (1 item) | How much was your life disrupted by the corona virus outbreak? Think about your daily routines, work, education and family. | 1=not at all to 10= extremely | --- |  |
| Economic disruption  (1 item) | I have financial problems because of the current situation (e.g. difficulties paying bills or rent). | 1= Yes and 0 = No | --- |  |
| Loss of occupation/education  (4 items) | How has the current corona crisis changed your occupational or educational situation?   - I was fired and am not working anymore/am looking for a job. - My educational program was suspended. - I am self-employed and have fewer customers/fewer orders. - I am self-employed and had to apply for interim government financial support. | 1= Yes and 0 = No | --- | Aggregated  (0= no item is endorsed, 1 = at least 1 item is endorsed) |
| Hopelessness  (1 item) | How hopeful are you when looking toward the future? | 1 = not at all hopeful to 10 = very hopeful (*reverse coded)* | --- | (possible range: 1-10) |
| Low trust in society’s response  (6 items) | The measures implemented by the authorities to handle the corona crisis are effective:   - I agree with the measures implemented for fighting the virus by the authorities. - The measures taken by the authorities against corona virus are fair. - I feel obligated to follow the rules implemented for fighting the virus. - The media inform about corona virus in a balanced and objective way. - Most people try to comply with the rules implemented for fighting the virus. | 1 = fully untrue to 4 = fully true (*reverse coded)* | 0.70 | Mean score  (possible range: 1-4) |
| Frequent COVID-19 news-seeking  (1 item) | On average, how often have you checked the news or looked for information regarding corona virus during the past two weeks? | 1=less than once a day and 5=pretty much constantly | --- | binary variable coded frequent news-seeking (i.e., 1 = “2-5 times a day” or “pretty much constantly”, 0 = less than that) |

| **Coping During COVID-19 (age 22)** | | | | |
| --- | --- | --- | --- | --- |
| Coping  -- adapted from Carver (1997) | *Item stem for all coping items:* There are lots of ways to try to deal with stress. The next few questions ask you to indicate how you dealt with stress in the past 2 weeks. There are no "right" or "wrong" answers. In the past two weeks, how often have you done the following things when you experienced something stressful? |  |  |  |
| *Emotional support seeking* | I have been seeking emotional support from others (e.g. from family, friends). | 1=not at all to 4=very frequently | *---* | (possible range: 1-4) |
| *Self-distraction* | I have been distracting myself with other things to take my mind off things (e.g. watching TV or films, reading, daydreaming, sleeping, playing with my pet, online shopping, cooking, cleaning, etc.) | 1=not at all to 4=very frequently | *---* | (possible range: 1-4) |
| *Acceptance* | I have been accepting the reality that the corona crisis is real. | 1=not at all to 4=very frequently | *---* | (possible range: 1-4) |
| *Positive Reappraisal/Reframing* | I have been trying to find something good in the corona crisis (e.g. that that due to the crisis my relationship with a person that is important to me has become stronger). | 1=not at all to 4=very frequently | *---* | (possible range: 1-4) |
| *Physical activity/exercise* | I have exercised or engaged in physical activity. | 1=not at all to 4=very frequently | *---* | (possible range: 1-4) |
| *Helping others* | I have been involved in helping neighbors during the corona crisis (e.g. went grocery shopping or picked up medicine for elderly/at-risk persons). | 1=not at all to 4=very frequently | *---* | dichotomized due to its highly skewed distributions (i.e., only a minority of participants reported that they had helped others).  (0 = not at all vs 1 = at least every now and then) |
| *Keeping in contact with family, friends, close others* | I have been keeping in contact with my family, other close persons and friends. | 1=not at all to 4=very frequently | *---* | (possible range: 1-4) |
| *Keeping daily routine* | I have been trying to keep up a daily routine. | 1=not at all to 4=very frequently | *---* | (possible range: 1-4) |
| *Seeking professional mental health support* | I have been seeking help at a telephone helpline, help chat, or from a counseling service or psychotherapist. | 1=not at all to 4=very frequently | *---* | dichotomized due to its highly skewed distributions (i.e., only a minority of participants reported any use of professional mental health support).  (0 = not at all vs 1 = at least every now and then) |

*Note.* The study questionnaire was administered in German. In instances where items were originally in English (but had been translated into German), we show the original English wording. For all other items, we show ad hoc translations of the original German items into English.

Supplementary Table S2. Descriptive statistics for all study variables by sex (based on weighted sample).

|  | Males | | | | | Females | | | | | |
| --- | --- | --- | --- | --- | --- | --- | --- | --- | --- | --- | --- |
|  | % | N | M | SD | % | | N | M | SD | *p* sex diff |  |
| **Outcomes at age 22, COVID-19** |  |  |  |  |  | |  |  |  |  |  |
| Perceived stress *(range: 1-5)* |  |  | 2.74 | 0.86 |  | |  | 3.10 | 0.94 | .000 |  |
| Internalizing symptoms *(range: 1-5)* |  |  | 1.85 | 0.62 |  | |  | 2.17 | 0.72 | .000 |  |
| Anger *(range: 1-5)* |  |  | 2.42 | 0.94 |  | |  | 2.76 | 0.91 | .000 |  |
| **Outcomes at age 20, pre-COVID-19** |  |  |  |  |  | |  |  |  |  |  |
| Perceived stress *(range: 1-5)* |  |  | 2.57 | 0.86 |  | |  | 3.02 | 0.98 | .000 |  |
| Internalizing symptoms *(range: 1-5)* |  |  | 1.91 | 0.61 |  | |  | 2.35 | 0.79 | .000 |  |
| Anger *(range: 1-5)* |  |  | 2.24 | 0.71 |  | |  | 2.51 | 0.78 | .000 |  |
| **Sociodemographics and living situation** |  |  |  |  |  | |  |  |  |  |  |
| Female | -- | -- | -- | -- | -- | | -- | -- | -- | -- |  |
| Family ISEI *(range: 10-90)* |  |  | 50.99 | 20.37 |  | |  | 50.07 | 19.05 | .517 |  |
| Migration background *(1=both parents born   abroad)* | 50.5 | 203/402 |  |  | 51.3 | | 192/374 |  |  | .815 |  |
| Education (age 20) |  |  |  |  |  | |  |  |  |  |  |
| Low (NEET) | 2.2 | 9/407 |  |  | 2.1 | | 8/377 |  |  | .932 |  |
| Medium | 70.0 | 285/407 |  |  | 69.2 | | 261/377 |  |  | .809 |  |
| High | 27.8 | 113/407 |  |  | 28.6 | | 108/377 |  |  | .784 |  |
| Living alone (age 22) | 4.7 | 19/402 |  |  | 6.2 | | 23/370 |  |  | .362 |  |
| **Stress and health before COVID-19 (age 20)** |  |  |  |  |  | |  |  |  |  |  |
| Perceived social exclusion *(range: 1-4)* |  |  | 1.45 | 0.54 |  | |  | 1.54 | 0.62 | .026 |  |
| Low social support *(range: 1-4)* |  |  | 1.75 | 0.67 |  | |  | 1.76 | 0.69 | .866 |  |
| Bullying victimization *(range: 1-6)* |  |  | 1.37 | 0.44 |  | |  | 1.36 | 0.49 | .690 |  |
| Low trust *(range: 1-4)* |  |  | 2.64 | 0.67 |  | |  | 2.66 | 0.67 | .775 |  |
| Sum of stressful life events in previous 3 years   *(range: 1-28)* |  |  | 3.64 | 2.44 |  | |  | 3.57 | 2.28 | .679 |  |
| Low self-rated health *(range: 0-100)* |  |  | 40.18 | 22.07 |  | |  | 47.64 | 23.13 | .000 |  |
| **Health risks during COVID-19 (age 22)** |  |  |  |  |  | |  |  |  |  |  |
| Occupational risks – loved one | 52.7 | 214/406 |  |  | 57.2 | | 215/376 |  |  | .209 |  |
| Health risks – loved one | 74.6 | 303/406 |  |  | 79.0 | | 297/376 |  |  | .150 |  |
| Actual illness – loved one | 13.1 | 53/406 |  |  | 15.2 | | 57/375 |  |  | .389 |  |
| Occupational risks – self | 27.1 | 110/406 |  |  | 25.8 | | 97/376 |  |  | .682 |  |
| Health risks – self | 33.0 | 134/406 |  |  | 34.6 | | 130/376 |  |  | .643 |  |
| Actual illness – self | 25.6 | 104/406 |  |  | 23.1 | | 87/376 |  |  | .420 |  |
| **Stressors during COVID-19 (age 22)** |  |  |  |  |  | |  |  |  |  |  |
| Lifestyle disruptions *(range: 1-10)* |  |  | 6.00 | 2.52 |  | |  | 6.58 | 2.33 | .001 |  |
| Economic disruption | 13.8 | 56/406 |  |  | 14.9 | | 56/375 |  |  | .650 |  |
| Loss of occupation/education | 7.6 | 31/408 |  |  | 5.6 | | 21/377 |  |  | .254 |  |
| Hopelessness *(range: 1-10)* |  |  | 3.86 | 1.98 |  | |  | 4.40 | 2.05 | .000 |  |
| Low trust in society’s response *(range: 1-4)* |  |  | 2.07 | 0.50 |  | |  | 2.03 | 0.44 | .159 |  |
| Frequent COVID-19 news-seeking | 28.2 | 115/408 |  |  | 29.1 | | 110/378 |  |  | .777 |  |

ISEI=International Socioeconomic Index of occupational status
NEET=not in education, employment, or training

Supplementary Table S3. Coefficients of association between pre- and during pandemic risk variables and emotional distress during COVID-19 as displayed in Figure 1 of the manuscript. Adjusted for sociodemographic variables. Risk variables were each entered one at a time (each risk variable=a separate model). Standardized regression coefficients (*β*).

|  | **Perceived Stress** | | | **Internalizing Symptoms** | | | **Anger** | |  |
| --- | --- | --- | --- | --- | --- | --- | --- | --- | --- |
|  | *β* | 95% CI | *p* | *β* | 95% CI | *p* | *β* | 95% CI | *p* |
| **Sociodemographics and living situation** |  |  |  |  |  |  |  |  |  |
| Sex (female) | **0.20** | 0.13 to 0.27 | <0.001 | **0.23** | 0.16 to 0.30 | <0.001 | **0.18** | 0.11 to 0.25 | 0.000 |
| Family ISEI | 0.04 | -0.05 to 0.13 | 0.396 | 0.00 | -0.09 to 0.09 | 0.943 | 0.03 | -0.06 to 0.12 | 0.572 |
| Migration background | **0.12** | 0.04 to 0.21 | 0.004 | ***0.08*** | 0.00 to 0.16 | 0.058 | 0.07 | -0.02 to 0.15 | 0.116 |
| Low education/occupation (NEET) | 0.00 | -0.08 to 0.07 | 0.815 | 0.04 | -0.03 to 0.11 | 0.217 | -0.01 | -0.08 to 0.05 | 0.702 |
| Medium education/occupation | -0.01 | -0.09 to 0.07 | 0.951 | 0.03 | -0.04 to 0.11 | 0.403 | -0.01 | -0.08 to 0.06 | 0.799 |
| Living alone (age 22) | 0.02 | -0.06 to 0.10 | 0.663 | **0.12** | 0.03 to 0.22 | 0.011 | 0.06 | -0.02 to 0.14 | 0.153 |
| **Stress and Health before COVID-19 (age 20)** |  |  |  |  |  |  |  |  |  |
| Perceived social exclusion | **0.29** | 0.22 to 0.36 | <0.001 | **0.37** | 0.30 to 0.44 | <0.001 | **0.26** | 0.19 to 0.33 | <0.001 |
| Low social support | **0.13** | 0.06 to 0.21 | 0.001 | **0.20** | 0.12 to 0.27 | <0.001 | **0.13** | 0.06 to 0.21 | 0.001 |
| Bullying victimization | **0.24** | 0.17 to 0.31 | <0.001 | **0.29** | 0.22 to 0.39 | <0.001 | **0.21** | 0.13 to 0.28 | <0.001 |
| Low trust | **0.14** | 0.07 to 0.22 | <0.001 | **0.19** | 0.12 to 0.26 | <0.001 | **0.22** | 0.15 to 0.30 | <0.001 |
| Sum of stressful life events in past 3 years | **0.19** | 0.13 to 0.26 | <0.001 | **0.19** | 0.11 to 0.26 | <0.001 | **0.17** | 0.10 to 0.24 | <0.001 |
| Low self-rated health | **0.22** | 0.16 to 0.29 | <0.001 | **0.25** | 0.17 to 0.32 | <0.001 | **0.18** | 0.11 to 0.26 | <0.001 |
|  |  |  |  |  |  |  |  |  |  |
| **Health risks during COVID-19 (age 22)** |  |  |  |  |  |  |  |  |  |
| Occupational risks–loved one | -0.01 | -0.08 to 0.07 | 0.892 | 0.01 | -0.06 to 0.08 | 0.756 | 0.05 | -0.02 to 0.12 | 0.159 |
| Health risks–loved one | **0.10** | 0.03 to 0.17 | 0.007 | **0.09** | 0.02 to 0.16 | 0.015 | 0.05 | -0.02 to 0.13 | 0.156 |
| Actual illness–loved one | **0.10** | 0.02 to 0.18 | 0.013 | ***0.07*** | -0.01 to 0.15 | 0.097 | 0.06 | -0.02 to 0.13 | 0.146 |
| Occupational risks–self | -0.02 | -0.10 to 0.05 | 0.501 | -0.01 | -0.08 to 0.07 | 0.904 | 0.02 | -0.06 to 0.09 | 0.690 |
| Health risks–self | **0.08** | 0.01 to 0.16 | 0.026 | **0.14** | 0.06 to 0.22 | 0.001 | **0.09** | 0.01 to 0.17 | 0.023 |
| Actual illness–self | **0.07** | 0.00 to 0.14 | 0.048 | **0.10** | 0.02 to 0.17 | 0.009 | **0.11** | 0.04 to 0.18 | 0.003 |
| **Stressors During COVID-19 (age 22)** |  |  |  |  |  |  |  |  |  |
| Lifestyle disruptions | **0.27** | 0.20 to 0.34 | <0.001 | **0.23** | 0.16 to 0.29 | <0.001 | **0.24** | 0.17 to 0.31 | <0.001 |
| Economic disruption | **0.18** | 0.10 to 0.26 | <0.001 | **0.27** | 0.19 to 0.35 | <0.001 | **0.17** | 0.09 to 0.24 | <0.001 |
| Loss of occupation/education | **0.11** | 0.03 to 0.18 | 0.005 | **0.14** | 0.06 to 0.23 | <0.001 | **0.12** | 0.05 to 0.19 | 0.002 |
| Hopelessness | **0.31** | 0.24 to 0.38 | <0.001 | **0.29** | 0.22 to 0.36 | <0.001 | **0.23** | 0.16 to 0.30 | <0.001 |
| Low trust in society’s response | **0.10** | 0.02 to 0.18 | 0.010 | **0.11** | 0.03 to 0.19 | 0.007 | **0.16** | 0.09 to 0.24 | <0.001 |
| Frequent COVID-19 news-seeking | **0.07** | 0.01 to 0.14 | 0.036 | 0.06 | -0.01 to 0.13 | 0.110 | **0.10** | 0.03 to 0.18 | 0.006 |

*Note.* ISEI=International Socioeconomic Index of occupational status; NEET=not in education, employment, or training. Coefficients with *p* < .05 in bold, *p*< .10 in bold and italics.

Supplementary Table S4. Coefficients of association between pre- and during pandemic risk variables and emotional distress during COVID-19 as displayed in Figure 2 of the manuscript. Adjusted for sociodemographic variables and the respective pre-pandemic emotional distress indicator. Risk variables were each entered one at a time (each risk variable=a separate model). Standardized regression coefficients (*β*).

|  | **Perceived Stress** | | | **Internalizing Symptoms** | | | **Anger** | | |
| --- | --- | --- | --- | --- | --- | --- | --- | --- | --- |
|  | *β* | 95% CI | *p* | *β* | 95% CI | *p* | *β* | 95% CI | *p* |
| **Sociodemographics and living situation** |  |  |  |  |  |  |  |  |  |
| Sex (female) | **0.10** | 0.03 to 0.16 | 0.005 | **0.07** | 0.01 to 0.14 | 0.028 | **0.12** | 0.05 to 0.19 | 0.001 |
| Family ISEI | 0.01 | -0.08 to 0.09 | 0.905 | -0.02 | -0.09 to 0.07 | 0.716 | 0.00 | -0.09 to 0.08 | 0.954 |
| Migration background | ***0.07*** | -0.01 to 0.15 | 0.074 | 0.04 | -0.04 to 0.11 | 0.318 | ***0.07*** | -0.01 to 0.15 | 0.087 |
| Low education/occupation (NEET) | 0.01 | -0.07 to 0.08 | 0.889 | -0.01 | -0.07 to 0.06 | 0.806 | -0.04 | -0.10 to 0.03 | 0.253 |
| Medium education/occupation | 0.00 | -0.07 to 0.08 | 0.951 | 0.02 | -0.05 to 0.08 | 0.604 | -.03 | -0.11 to 0.04 | 0.341 |
| Living alone (age 22) | -0.02 | -0.09 to 0.05 | 0.625 | 0.06 | -0.02 to 0.14 | 0.155 | 0.03 | -0.05 to 0.11 | 0.500 |
|  |  |  |  |  |  |  |  |  |  |
| **Outcomes at age 20** | **0.42** | 0.35 to 0.48 | <0.001 | **0.53** | 0.46 to 0.59 | <0.001 | **0.32** | 0.25 to 0.39 | <0.001 |
|  |  |  |  |  |  |  |  |  |  |
| **Stress and Health before COVID-19 (age 20)** |  |  |  |  |  |  |  |  |  |
| Perceived social exclusion | **0.10** | 0.03 to 0.18 | 0.007 | 0.07 | -0.02 to 0.16 | 0.134 | **0.15** | 0.08 to 0.23 | <0.001 |
| Low social support | 0.04 | -0.04 to 0.11 | 0.337 | 0.05 | -0.02 to 0.13 | 0.146 | ***0.07*** | 0.00 to 0.15 | 0.058 |
| Bullying victimization | **0.11** | 0.05 to 0.18 | 0.001 | **0.10** | 0.03 to 0.17 | 0.004 | **0.10** | 0.03 to 0.18 | 0.008 |
| Low trust | 0.04 | -0.04 to 0.11 | 0.337 | 0.04 | -0.03 to 0.11 | 0.218 | **0.15** | 0.08 to 0.23 | <0.001 |
| Sum of stressful life events in past 3 years | **0.11** | 0.05 to 0.18 | 0.001 | **0.08** | 0.01 to 0.16 | 0.020 | **0.11** | 0.05 to 0.18 | 0.001 |
| Low self-rated health | **0.10** | 0.04 to 0.17 | 0.003 | **0.07** | 0.00 to 0.14 | 0.039 | **0.12** | 0.05 to 0.19 | 0.001 |
|  |  |  |  |  |  |  |  |  |  |
| **Health risks during COVID-19 (age 22)** |  |  |  |  |  |  |  |  |  |
| Occupational risks–loved one | -0.02 | -0.08 to 0.05 | 0.561 | 0.00 | -0.06 to 0.06 | 0.923 | 0.03 | -0.04 to 0.10 | 0.331 |
| Health risks–loved one | **0.07** | 0.01 to 0.14 | 0.034 | ***0.06*** | 0.00 to 0.12 | 0.061 | 0.04 | -0.03 to 0.11 | 0.212 |
| Actual illness–loved one | **0.09** | 0.02 to 0.16 | 0.012 | ***0.06*** | 0.00 to 0.13 | 0.064 | 0.06 | -0.02 to 0.13 | 0.136 |
| Occupational risks–self | -0.04 | -0.11 to 0.02 | 0.193 | -0.02 | -0.09 to 0.05 | 0.563 | 0.01 | -0.06 to 0.08 | 0.810 |
| Health risks–self | 0.05 | -0.02 to 0.12 | 0.125 | **0.08** | 0.01 to 0.16 | 0.028 | ***0.07*** | -0.01 to 0.14 | 0.090 |
| Actual illness–self | 0.03 | -0.04 to 0.09 | 0.416 | 0.03 | -0.04 to 0.09 | 0.459 | **0.08** | 0.01 to 0.15 | 0.036 |
|  |  |  |  |  |  |  |  |  |  |
| **Stressors during COVID-19 (age 22)** |  |  |  |  |  |  |  |  |  |
| Lifestyle disruptions | **0.24** | 0.18 to 0.31 | <0.001 | **0.21** | 0.15 to 0.27 | <0.001 | **0.25** | 0.18 to 0.32 | <0.001 |
| Economic disruption | **0.14** | 0.08 to 0.21 | <0.001 | **0.24** | 0.17 to 0.31 | <0.001 | **0.17** | 0.10 to 0.24 | <0.001 |
| Loss of occupation/education | **0.07** | 0.01 to 0.13 | 0.035 | **0.11** | 0.05 to 0.18 | 0.001 | **0.11** | 0.04 to 0.18 | <0.001 |
| Hopelessness | **0.24** | 0.17 to 0.30 | <0.001 | **0.18** | 0.11 to 0.25 | <0.001 | **0.20** | 0.12 to 0.27 | <0.001 |
| Low trust in society’s response | **0.08** | 0.01 to 0.15 | 0.024 | **0.07** | 0.00 to 0.14 | 0.049 | **0.15** | 0.08 to 0.22 | <0.001 |
| Frequent COVID-19 news-seeking | ***0.06*** | -0.01 to 0.12 | 0.083 | ***0.05*** | -0.01 to 0.11 | 0.086 | **0.10** | 0.03 to 0.17 | <0.001 |

*Note.* ISEI=International Socioeconomic Index of occupational status; NEET=not in education, employment, or training. Coefficients with *p* < .05 in bold, *p*< .10 in bold and italics.

Supplementary Table S5. Descriptive statistics of coping variables (based on weighted sample).

|  | % | N | M | SD |
| --- | --- | --- | --- | --- |
| Emotional support seeking (*range: 1-4)* |  |  | 2.07 | 0.89 |
| Self-distraction (*range: 1-4)* |  |  | 2.90 | 0.88 |
| Acceptance (of COVID-19 crisis) (*range: 1-4)* |  |  | 3.14 | 0.83 |
| Positive reappraisal/reframing (*range: 1-4)* |  |  | 2.66 | 1.04 |
| Physical activity/exercise (*range: 1-4)* |  |  | 2.54 | 1.05 |
| Helping others (e.g., in neighborhood) | 32.6 | 256 |  |  |
| Keeping contact with family/friends (*range: 1-4)* |  |  | 2.98 | 0.83 |
| Keeping daily routine (*range: 1-4)* |  |  | 2.76 | 0.90 |
| Seeking professional help | 7.7 | 61 |  |  |

**References**

Bude, H., & Lantermann, E. D. (2006). Soziale Exklusion und Exklusionsempfinden [Social exclusion and feeling socially excluded]. *Kölner Zeitschrift für Soziologie und Sozialpsychologie, 25*, 233-252.

Carver, C. S. (1997). You want to measure coping but your protocol's too long: consider the brief COPE. *International Journal of Behavioral Medicine, 4*(1), 92-100. doi:10.1207/s15327558ijbm0401_6

Cohen, S., Kamarck, T., & Mermelstein, R. (1983). A global measure of perceived stress. *Journal of Health and Social Behavior, 24*, 385-396.

Inglehart, R., Haerpfer, C., Moreno, A., Welzel, C., Kizilova, K., Diez-Medrano, J., . . . Puranen, B. (2014). World Values Survey: All Rounds - Country-Pooled Datafile Version: <http://www.worldvaluessurvey.org/WVSDocumentationWVL.jsp:> JD Systems Institute.

Murray, A. L., Eisner, M., Ribeaud, D., Kaiser, D., McKenzie, K., & Murray, G. (2019). Validation of a brief self-report measure of adolescent bullying perpetration and victimization. *Assessment*, 1073191119858406. doi:10.1177/1073191119858406

Murray, A. L., Obsuth, I., Eisner, M., & Ribeaud, D. (2019). Evaluating longitudinal invariance in dimensions of mental health across adolescence: An analysis of the Social Behavior Questionnaire. *Assessment, 26*(7), 1234-1245. doi:10.1177/1073191117721741

Pilkonis, P. A., Choi, S. W., Reise, S. P., Stover, A. M., Riley, W. T., Cella, D., & Group, P. C. (2011). Item banks for measuring emotional distress from the Patient-Reported Outcomes Measurement Information System (PROMIS(R)): depression, anxiety, and anger. *Assessment, 18*(3), 263-283. doi:10.1177/1073191111411667
